# Low Aflatoxin Levels in *Aspergillus flavus*-Resistant Maize Are Correlated With Increased Corn Earworm Damage and Enhanced Seed Fumonisin

**DOI:** 10.3389/fpls.2020.565323

**Published:** 2020-09-23

**Authors:** Subbaiah Chalivendra, Fangneng Huang, Mark Busman, W. Paul Williams, Jong Hyun Ham

**Affiliations:** ^1^ Department of Plant Pathology and Crop Physiology, Louisiana State University AgCenter, Baton Rouge, LA, United States; ^2^ Department of Entomology, Louisiana State University AgCenter, Baton Rouge, LA, United States; ^3^ Bacterial Foodborne Pathogens and Mycology Research Unit, USDA-ARS-NCAUR, Peoria, IL, United States; ^4^ Corn Host Plant Resistance Research Unit, USDA-ARS, Mississippi State, MS, United States

**Keywords:** Maize (*Zea mays*), *Aspergillus flavus*, *Fusarium verticillioides*, ear rot, resistant and susceptible lines, corn earworm (*Helicoverpa zea* Boddie), aflatoxin toxicity, fumonisin tolerance

## Abstract

Preharvest mycotoxin contamination of field-grown crops is influenced not only by the host genotype, but also by inoculum load, insect pressure and their confounding interactions with seasonal weather. In two different field trials, we observed a preference in the natural infestation of corn earworm (CEW; *Helicoverpa zea* Boddie) to specific maize (*Zea mays* L.) genotypes and investigated this observation. The field trials involved four maize lines with contrasting levels of resistance to *Aspergillus flavus*. The resistant lines had 7 to 14-fold greater infested ears than the susceptible lines. Seed aflatoxin B_1_ (AF) levels, in mock- and *A. flavus*-inoculated ears were consistent with genotype resistance to *A. flavus*, in that the resistant lines showed low levels of AF (<30 ppb), whereas the susceptible lines had up to 500 ppb. On the other hand, CEW infestation showed a positive correlation with seed fumonisins (FUM) contamination by native *Fusarium verticillioides* strains. We inferred that the inverse trend in the correlation of AF and FUM with *H. zea* infestation may be due to a differential sensitivity of CEW to the two mycotoxins. This hypothesis was tested by toxin-feeding studies. *H. zea* larvae showed decreasing mass with increasing AF in the diet and incurred >30% lethality at 250 ppb. In contrast, CEW was tolerant to fumonisin with no significant loss in larval mass even at 100 ppm, implicating the low seed aflatoxin content as a predominant factor for the prevalence of CEW infestation and the associated fumonisin contamination in *A. flavus* resistant maize lines. Further, delayed flowering of the two resistant maize lines might have contributed to the pervasive *H. zea* damage of these lines by providing young silk for egg-laying. These results highlight the need for integrated strategies targeting mycotoxigenic fungi as well as their insect vectors for enhanced food safety.

## Introduction

Besides causing crop damage and economic loss to the grower, mycotoxigenic fungi pose a serious risk to human and livestock health due to the contamination of commodities with carcinogenic and neurotoxic secondary metabolites known as mycotoxins. Aflatoxin B_1_ (AF) is the most dangerous among mycotoxins due to its very potent carcinogenicity. *Aspergillus flavus*, an opportunistic pathogen, is the predominant species that contaminates cereal and oil seed crops with AF. Although not as genotoxic as AF, fumonisins (FUM) are associated with esophageal cancer, particularly due to cytotoxicity of fumonisin B_1_ (FB_1_). They are also among the most common food- and feed-contaminating mycotoxins in many countries (Biomin, 2015; Munkvold et al., 2019). FUM are produced by *Fusarium* species*, F. verticillioides* (formerly known as *F. moniliforme*) being the predominant contaminant of commodities (Munkvold, 2003). *A. flavus* and *F. verticillioides* cause ear rots in maize (*Zea mays* L.), a globally important food, feed and fuel crop of high productivity. AF and FUM can be co-contaminants of commodities (Guo et al., 2017), particularly in high cancer-risk areas (Sun et al., 2011), and act synergistically on carcinogenesis (Lopez-garcia, 1998).

Aspergillus and Fusarium ear rots are more frequent in warmer and drier cropping seasons or a warmer and wetter weather combination at the time of harvest, and are often exacerbated by insect damage. Insect-vectored inoculum can breach the natural plant defense. The invasive methods of inoculation by chewing and piercing insects would bypass resistance mechanisms, such as remote defense signals triggered in the husk, silk or seed surface in response to natural infection *via* silk. Consequently, ear rot diseases are more common in the southern United States (US) and lowland tropics (Miller, 1994; reviewed in Cotty and Jaime-Garcia, 2007; Santiago et al., 2015). Among insect pests infesting maize, European Corn Borer (ECB) causes the most serious damage (Boyd and Bailey, 2001; Hutchison et al., 2010). It not only injures plants, exposing them to infection, but also vectors ear rot and stalk rot fungi, particularly *F. verticillioides* and *F. graminearum* (Widstrom, 1992). Extensive use of Bt (*Bacillus thurigiensis* crystal proteins-expressing) maize, with its high efficacy against ECB, has reduced overall ECB populations in the US (Hutchison et al., 2010). Maize pests previously considered as secondary to ECB are now taking its position (Bowers et al., 2014). Corn earworm [CEW; *Helicoverpa zea* (Boddie); formerly in the genus *Heliothis*] has become the most economically important pest in the southern US where non-freezing winters are conducive for it to multiply by 4–7 generations in a year. Resistance of this pest to a wide range of insecticides and to Bt maize has also been documented (Capinera, 2004; Dively et al., 2016; Kaur et al., 2019). Although CEW has multiple crop and weed hosts, maize is its preferred host (Johnson et al., 1975). Annual yield loss due to CEW ranges from 2–17% for field corn and up to 50% in sweetcorn in the southern US. *A. flavus* and *F. verticillioides* invade the seed through silk and are also vectored by CEW and other ear-infesting insects (Munkvold and White, 2016). *F. verticillioides* can grow also as an endophyte through root or stem infection, and is vectored by insects such as ECB that feed on vegetative tissues (Blacutt et al., 2018). In addition to facilitating fungal colonization, insect infestation can also enhance mycotoxin production in host tissues (Döll et al., 2013; Drakulic et al., 2015; Drakulic et al., 2016). Unlike a strong association observed in the case of FUM contamination (e.g., Smeltzer, 1959; Dowd, 2000; Mesterházy et al., 2012), seed AF levels were reported to be poorly correlated with CEW damage caused by either natural invasion (Ni et al., 2011; Cao et al., 2014) or manual infestation (Lillehoj et al., 1984). A meta-analysis of published work showed a 59% reduction in the mean FB_1_ concentration in Bt maize compared to the non-Bt control (Cappelle, 2018).

Insect–fungal interactions are much more complex than vector–cargo relationships and have domino effects on host colonization (Schulthess et al., 2002; Ako et al., 2003; Piesik et al., 2011; Döll et al., 2013; Drakulic et al., 2015; Drakulic et al., 2016). For example, AF is known to be toxic to CEW based on *in vitro* studies (Zeng et al., 2006). Recent work on *Drosophila* further suggests that aflatoxigenic *A. flavus* strains may have greater fitness than non-aflatoxigenic strains in the presence of insects (Drott et al., 2017). However, there has been no study on the implications of these observations in the context of AF production in crop hosts. It was fortuitous that we observed a preferential CEW infestation and increased FUM contamination in *A. flavus* resistant maize lines in two unrelated field trials. These observations were robust and derived from two different sets of resistant and susceptible maize lines (details in the *Materials and Methods* section). Since it is relevant to mycotoxin mitigation, we pursued to unravel the factors underlying this novel host–pathogen–insect interaction. Late flowering might have facilitated enhanced oviposition by *H. zea* in the resistant maize lines, but our analysis suggests that the toxicity of AF to CEW is a more compelling reason for the observed prevalence of ear damage in the low AF accumulating genotypes.

## Materials and Methods

### Maize Field Trials Related to the Study

Field experiments were carried out at the Agricultural Research Station, LSU AgCenter, Baton Rouge. The four maize genotypes used in both trials are non-transgenic and non-commercial lines. The first or “hybrid” study used two hybrids, GA209 × T173 (susceptible to AF accumulation) and Mp313E × Mp717 (resistant to AF accumulation) that were developed at the USDA-ARS Corn Host Plant Resistance Research Unit, Mississippi (Williams and Windham, 2009). Toxigenic *A. flavus* strain, CA14 (obtained from the USDA Agricultural Research Service Culture Collection, Northern Regional Research Laboratory, Peoria, IL, USA) was used in the study. The strain has whole genome sequence information and needed mutant resources (Chang et al., 2019). The second or “inbred” study was done with two popular inbreds B73 (susceptible to AF accumulation, (Campbell and White, 1995) and CML322 (resistant to AF accumulation, (Betrán et al., 2002). Tox4, an isolate from local maize fields (Chalivendra et al., 2018), was used in the study because it is produces high AF levels and serves as a good model strain to study microbiome changes, which is the planned objective of the study.

All four lines were planted in 4-row plots in the middle of April, 2018. To keep the insect pressure low, Besiege, a broad-spectrum foliar insecticide with chlorantraniliprole and λ-cyhalothrin as active ingredients, was sprayed at ~V9 and R1 growth stages. Three days after the second insecticide application, plants were inoculated with conidial suspensions of *A. flavus* strains by silk canal injections, as described before (Zummo and Scott, 1992). Plants were maintained with standard agronomic practices of fertilizer and herbicide applications and received irrigations during extended dry periods.

### Weather Data

The 2018 cropping season in the US was unusual in its weather pattern. Daily high and low temperatures and rainfall data were downloaded from https://www.wunderground.com/history/monthly/us/la/baton-rouge/KBTR/ for April to July months of maize cropping season in 2017 and 2018 and are shown in **Figure S1**.

### Assessment of Earworm Damage and Mycotoxin Measurements

One ear per plant from each genotype and treatment was harvested, resulting in 70–80 ears in inoculated plants and double the number from uninoculated plants. Ears in each lot were separated by the presence or absence of CEW infestation to monitor the effect of insect damage on mycotoxin levels. Only ears with visible internal damage (i.e., nibbled seed and cut silks, larval feeding tracks with frass; sometimes with dead or live CEW larvae) were considered as infested. No distinct spatial or other pattern of infestation was observed in our plots (also see Ni et al., 2011), except that a majority of resistant inbred or hybrid plants were infested, while only a few ears from susceptible lines showed damage by the earworm. At least three ears were used per replicate and each category had 3–5 replicates. Given the low frequency of CEW-damaged ears in B73 and GA209 × T173, all ears in each category were used for AF analysis to have robust AF data. When the seed meal exceeded more than 100 g (in uninoculated controls), we took more than one sample to minimize sampling error.

AF from seed meal was extracted and measured as before (Chalivendra et al., 2018) using modified high performance liquid chromatography (HPLC) conditions. The equipment included Waters e2695 HPLC (Waters Corp., Milford, MA, United States) fitted with a Nova-Pak C18 column, a photochemical reactor (Aura Industries Inc., New York, United States) and a Waters 2475 FLR Detector (Waters Corp.). The signal was detected by excitation at 365 nm and emission at 440 nm. Aqueous methanol (37.5%) was used as the mobile phase.

FB_1_, FB_2_, and FB_3_ in the same maize seed meal samples were analyzed by liquid chromatography–mass spectrometry (LC-MS) using an adaptation of a previously published method for mycotoxin analysis (Plattner, 1999). Briefly, maize samples were ground with a laboratory mill. Portions (5 g) of the seed meal were extracted with 25 mL 1:1 acetonitrile/water for 2 h on a Model G2 Gyrotory Shaker (New Brunswick Scientific, Edison, NJ, USA). Extracts were filtered with a Whatman 125 mm 2V paper filter (GE Healthcare Bio-Sciences, Pittsburgh, PA, USA). A total of 10 µL of extract was applied to a Kinetex (Phenomenex, Torrance, CA, USA) C18 column (50 mm length, 2.1 mm diameter). Chromatography was conducted utilizing a Thermo Dionex Ultimate 3000 (Thermo Fisher, Waltham, MA, USA) ultrahigh-performance liquid chromatography (UPLC) system consisting of an autosampler coupled to a binary gradient pump. Elution of analyte was achieved with a 0.6 mL min^−1^ gradient flow of methanol and water (0.3% acetic acid was added to the mobile phase). The solvent program used a 35–95% gradient over 5 min. Flow was directed to a Q Exactive (Thermo Fisher, Waltham, MA, USA) hybrid quadrupole-Orbitrap mass spectrometer equipped with an electrospray ionization source. The mass spectrometer was operated in full-scan mode over a range of 300 to 1,200 m/z. Operation of the LC-MS and quantification of the eluting fumonisins were performed utilizing Thermo Xcalibur software. Quantification of fumonisins was based upon intensity of protonated ions for FB_1_ (m/z 722.3), FB_2_ (m/z 706.3) and FB_3_ (m/z 706.3) compared to calibration standards of the toxins. The limit of quantification for the analytical method was determined to be 0.1 µg per g for FB_1_, FB_2_, and FB_3_.

### Bioassays for Mycotoxin Toxicities to CEW

The toxicities of AF and FUM to CEW larvae were tested in a pre-mixed meridic diet (WARD’S Stonefly Heliothis diet, Rochester, NY) by supplementing with 0, 3, 10, 30 60, or 100 μg/g FB_1_ (Cayman Chemical, MI) or 0, 20, 50, 100, 250, or 500 ng/g of AFB_1_ (Sigma Chemicals). The diet was prepared as per manufacturer’s instructions. The FB_1_ stock, made in water, was diluted to the above rates before the dry diet was added and mixed thoroughly. AF was dissolved in methanol at a stock concentration of 2 mg/mL and diluted appropriately to provide the aforementioned concentrations. The highest concentration of methanol used (0.08% by w/w) was incorporated into the control diet. The assay was done in a 128 well bioassay plate (C-D International Inc., Pitman, NJ). A single CEW neonate from a laboratory CEW colony obtained from Benzon Research Inc. (Carlisle, PA) was added to each well with 1 g of diet using a camel hair brush (Kaur et al., 2019). At least 20 larvae were tested per treatment and the assay was repeated four times.

### Statistical Analysis of Data

Insect damage and aflatoxin levels were compared by ANOVA and post-hoc analysis by Tukey’s Honestly Significant Difference (HSD) test using R program (version 3.6.2) in RStudio. Student’s t-test was used for comparison of specific pairs of data sets.

### Safety

AF and FB_1_, being highly toxic mycotoxins, were handled with care using a biohood, surgical gloves and nose as well as mouth masks. All residues and containers were decontaminated using bleach and by autoclaving.

## Results

### Corn Earworm Outbreak in 2018 Summer

The unexpected observation that prompted the current work was made in two separate field experiments in 2018. The objective of hybrid study was to correlate the transcripts of *A. flavus* *medusa A* gene with the spatial distribution of the biofilm-like structure in maize seeds. Previous studies showed that *A. flavus* forms biofilm-like structure during maize seed colonization (Dolezal et al., 2013; Shu et al., 2014; Windham et al., 2018). The aim of the inbred study was to analyze microbiome changes in the susceptible and a resistant line in response to *A. flavus* colonization.

During the summer of 2018, daily profiles of rain fall and air temperature patterns were different from past years’ average in Louisiana as well as many of the maize-growing states in US. The growing season was shorter (late April to early August) due to extended cold temperatures into the beginning of the planting season and relatively warmer and drier days during the early crop growth period (**Figure S1**). April 2018 was the coldest April month since 1997 based on US average temperatures (and for Iowa and Wisconsin, it was the coldest April since records began in 1895). In contrast, May 2018 was the hottest May on record, breaking the record set in May 1934 during the Dust Bowl (National Oceanic and Atmospheric Administration: https://www.noaa.gov/). The unseasonal and steep warming, and the dry weather after protracted cold seems to have favored an explosion of CEW population as indicated by a heavy infestation of ears in both of our experimental plots. CEW incidence was also reported from maize fields in other states in southern (Porter and Bynum, 2018) as well as northern US (e.g., Handley, 2018). A similar buildup of CEW reported in Michigan in 2019 was also attributed to unusual weather pattern (Schuh and Springborn, 2019). In spite of two applications (before and after silking) of a strong broad-spectrum insecticide with fast knockdown as well as long-lasting residual effects, the insecticide seems to have failed to reach silks covered by the husks. Further, all ears were bagged immediately after inoculation/pollination, which concealed earworm damage until developing ears were sampled for analysis.

### CEW Infestation Was Significantly Greater in *A. flavus* Resistant Maize Lines

**Figure 1:**
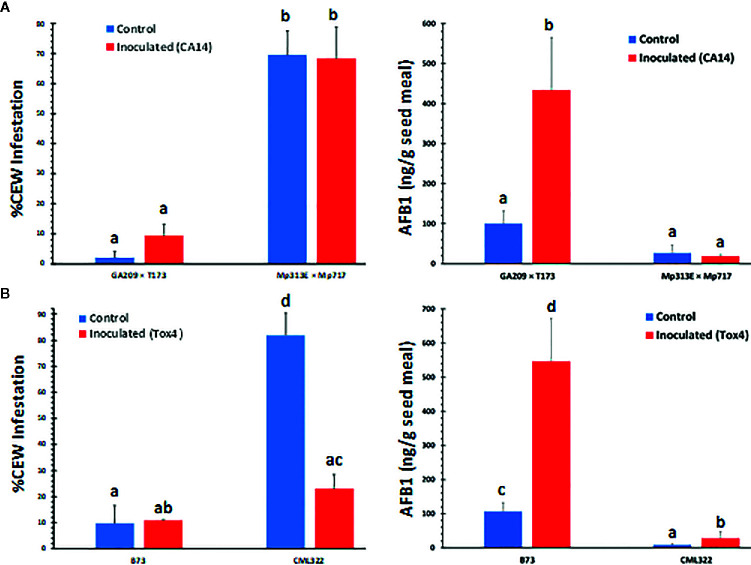
Rate of corn earworm infestation (left panels) and seed AF content (right panels) in maize lines. **(A)** Data is from hybrid plots. Infestation was significantly dependent on the host genotype with very little difference between control (mock-inoculated) and CA14-inoculated set. **(B)** Data shown is from inbreds. Values shown are average + SE. Significant differences (P value <0.05) between each data set were tested using an ANOVA (**Table S1**) followed by Tukey’s multiple-comparisons *post hoc* test in R (version 3.6.2). Means are significantly different if marked by a different letter.

During sampling of ears later in the season (July), we noticed that the two resistant lines, the hybrid Mp313E × Mp717 and the inbred CML322 showed greater infestation by CEW than the susceptible lines GA209 × T173 and B73 (**Figure 1**, left panels). The infestation was <10% in susceptible lines and it ranged from 22 to 68% in the resistant lines. The maize lines used in the two field trials have been extensively validated in the field and are often used as checks for evaluating new genotypes and in mapping resistance loci (e.g., Mideros et al., 2012; Guo et al., 2017). Despite our concerns that the distinctive patterns of CEW infestation might potentially interfere with the genetic response of maize lines to *A. flavus*, AF measurements showed that the genotype responses were robust in spite of CEW infestation. As described in the *Materials and Methods* section, we harvested and utilized all ears in the plots to obtain robust AF data. The insect infestation was 8-fold greater in CML322 than observed in B73 ears in the mock-inoculated set. Inoculation with the highly toxigenic Tox4 strain resulted in a significant (p<0.01) and nearly 4-fold decrease in the infestation of CML322, but still 2-fold greater than infestation in B73. This is inversely correlated with >3-fold increase in seed AF content in Tox4- inoculated CML322 ears. As expected from its susceptibility to *A. flavus* colonization, B73 seeds accumulated >100 ppb of AF even in mock-inoculated (Control) ears and >500 ppb in Tox4-inoculated ears. These AF levels are >12–19 fold higher than those measured in CML322 seeds (**Figure 1B**, right panel). CEW infestation was also greater in the resistant hybrid (Mp313E × Mp717) than in the susceptible hybrid by >30-fold in the control set and by 7-fold in the inoculated set (**Figure 1A**, left panel). Infestation was inversely correlated with seed AF levels in hybrids as well. The susceptible hybrid (GA209 × T173) had 100 ppb of AF in uninoculated control seeds and >400 ppb in the inoculated set (i.e., 3 and 24-fold greater than in the resistant hybrid). Unlike the resistant inbred CML322, the resistant hybrid showed no difference in either AF content or CEW infestation between the control and CA14-inoculated ears. Analysis of variance (ANOVA) confirmed that only the host genotype (i.e., resistance to *A. flavus*) affected infestation highly significantly (>99.99% confidence level) and inoculation-induced differences were not statistically different (**Table S1**).

### CEW Infestation Is Negatively Correlated With Seed AF Content

**Figure 2:**
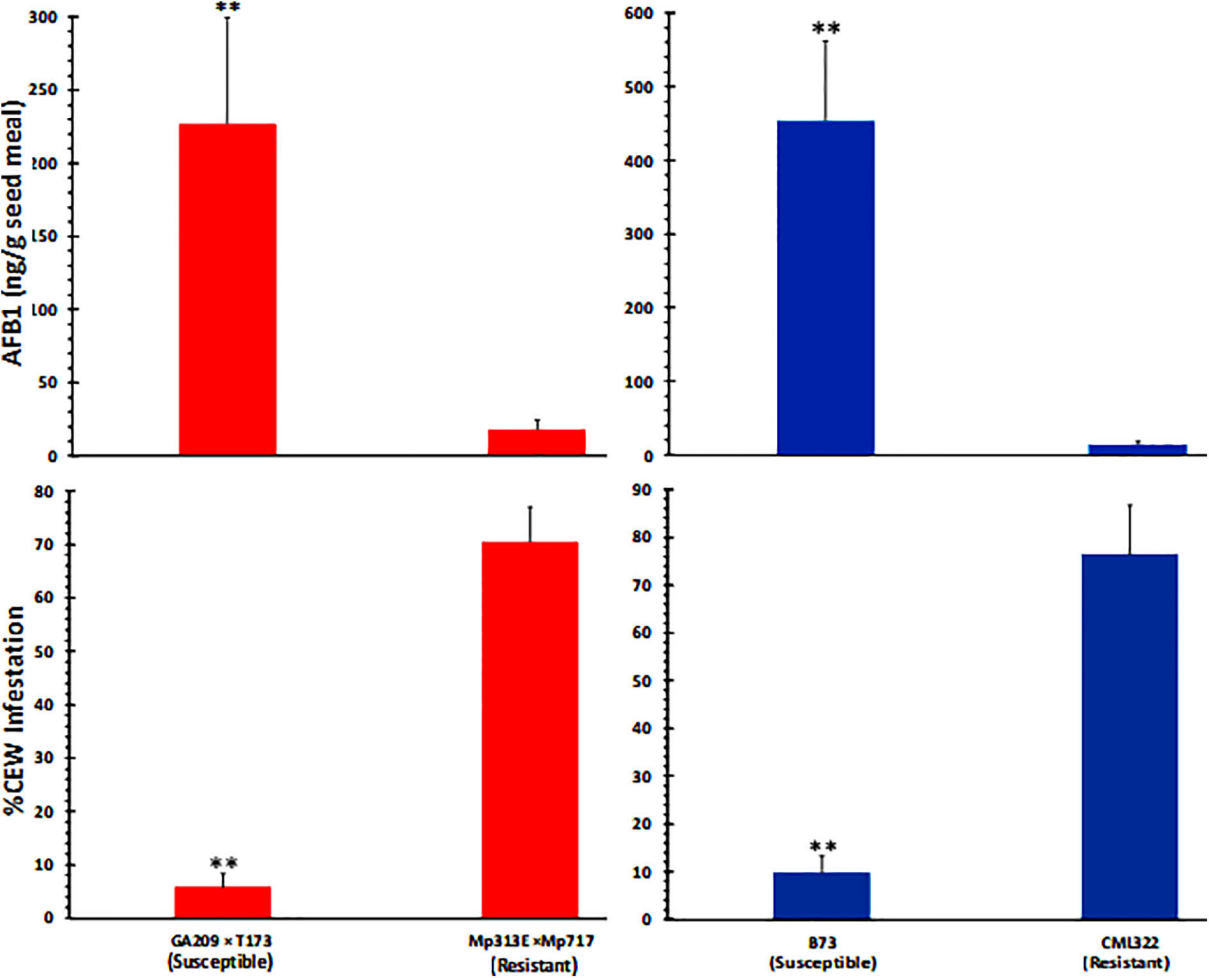
CEW damage is negatively correlated with seed AF content in maize 20 lines. The infestation and AF data from control and infected ears is combined in each genotype. Significant differences (P value <0.05) between each data set were tested using an ANOVA followed by Tukey’s multiple-comparisons *post hoc* test in R. Average (+SE) infestation and AF values between *A. flavus* susceptible and resistant lines are highly significant (denoted by **;p < 0.01).

As can be expected from the above data (**Figure 1**), ANOVA of seed AF content across the two experiments (**Table S2**) revealed that the host genotype and inoculation with toxigenic *A. flavus* strains showed highly significant direct as well as interaction effects on seed AF content. Infestation was also significantly related to AF content, although its interaction effect with genotype on AF was not significant. Both the resistant genotypes (CML322 and Mp313E × Mp717) manifested robust resistance to *A. flavus* and accumulated less than 30 ppb of AF in the seed either in the control (via colonization of native *A. flavus* strains) or the inoculated set. Conversely, the susceptible inbred and hybrid accumulated 100 and 500 ppb in control and inoculated sets, respectively. AF content is inversely correlated with CEW infestation pattern in each of the four maize genotypes. This relationship becomes clear when the data is combined for control and inoculated sets in each genotype (**Figure 2**) or when all data is combined (**Figure S2**). It is of interest to note that the uninfected controls from both resistant lines showed a numerical but statistically insignificant increase in AF in CEW-infested ears. AF was scarcely detectable in the uninfested and uninoculated controls (a mean value of 6 ppb in Mp313E × Mp717 and <1 ppb in CML322) but increased by 5 and 14-fold in infested ears of resistant hybrid and inbred respectively. This suggested that resistance to AF contamination might have been compromised to some extent in seeds heavily damaged by CEW.

### Kernel Fumonisin Content Was Enhanced in CEW-Infested Ears

**Figure 3:**
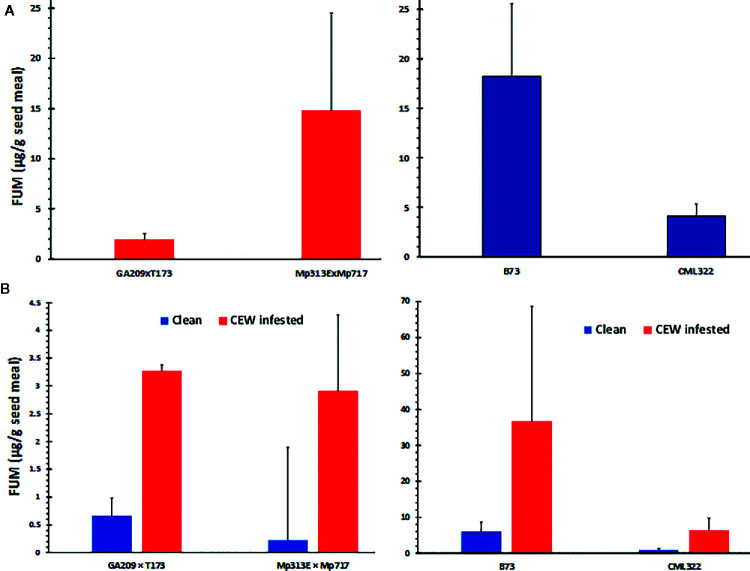
FUM contamination by native *Fusarium* strains. **(A)** Seed fumonisin content in the four maize lines. **(B)** Seed FUM content parsed by uninfested (clean) versus CEW infested ears in each genotype. The values are averages + SE in each genotype and were not significantly different at 95% confidence level.

*Fusarium verticillioides* is among the most common mycotoxigenic fungi colonizing field-grown maize. We observed symptoms of *F. verticillioides* colonization (e.g., star-burst pattern on seeds) in our samples. We isolated the fungus from seeds with visual symptoms using *Fusarium*-selective Malachite Green Agar 2.5 medium (Alborch et al., 2010) and confirmed the species identity by genomic PCR using *F*. *verticillioides*-specific primers (Baird et al., 2008). FUM content was analyzed in the same seed samples used for AF determination (**Figure 3A**) and compared between uninfested and CEW-infested samples (**Figure 3B**).

Both maize hybrids used in this study have been previously shown to be resistant to FUM accumulation. The *A. flavus* resistant hybrid, Mp313E × Mp717 was shown to be more robustly resistant than GA209 × T173 in multiple studies (Williams, 2006; Henry et al., 2009; Williams and Windham, 2009). In the current study, however, the Mp313E × Mp717 accumulated >7-fold greater concentration of FUM in its seeds than GA209 × T173 (**Figure 3A**). Although CML322 accumulated a considerable amount of FUM, it was >4-fold less than that in B73, which is known to be among the most susceptible inbreds to Fusarium ear rot and FUM accumulation (Morales et al., 2019). However, when the data was parsed based on CEW infestation (only in sets where both clean and infested ears were available), infested ears showed >5-fold more FUM than uninfested ears (**Figure 3B**). The differences were not significant probably due to the high variability in the colonization by native strains (the lowest p-value was 0.052 for CML322; also see **Figure S2**). These data indicated that CEW may vector *Fusarium* spp. that produce FUM during its infestation, as often reported in the literature (Munkvold et al., 2019).

### Differential Toxicity of AF Versus FB_1_ to CEW

**Figure 4:**
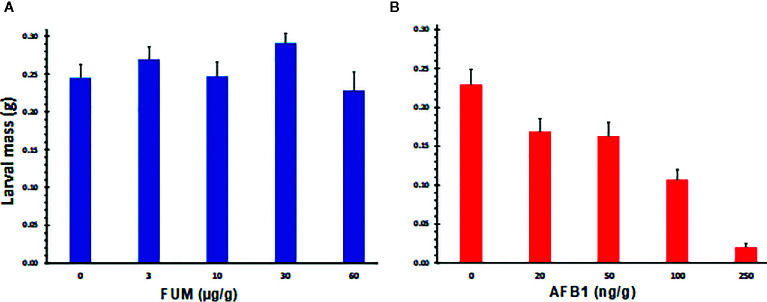
Effects of aflatoxin B_1_ and fumonisin B_1_ on the growth of *H. zea* larvae, as measured by the body mass. Graded doses of FB_1_ and AF (**A**, **B** respectively) were tested on CEW growth and mortality by incorporating them into an artificial insect diet. Larvae were grown in a 128 well bioassay plate for 10 d. Each well had 1 g of feed and a single neonate at the start of the assay. A representative assay from 4 replicated experiments is shown (**Figure S3**). At the end of the bioassay, larvae were removed from the well killed by chloroform vapors and weighed. Values are averages + SE of ≥16 larvae/treatment except at 250 ppb of AF, where mortality was 30% or greater (dead and dried larvae were seen stuck to the bottom of the well). The values marked with the same letter are not statistically significant. FB_1_ had no significant effect on larval growth at concentrations tested.

The preferential infestation of *A. flavus* resistant lines by CEW, the negative correlation between AF and CEW infestation levels, and a greater FUM levels in infested ears, suggested that AF may be more toxic to *H. zea* than FUM. We tested this hypothesis by feeding experiments where CEW neonates were reared on artificial diet containing graded levels of AF or FB_1_. Results shown in **Figure S3** and **Figures 4A, B** clearly demonstrate that the pest is more susceptible to AF than to FB_1_. As reported previously (Zeng et al., 2006), AF retarded CEW larval growth even at the lowest concentration tested, although the effect was not significant (**Figure 4B**) and was lethal above 200 ppb (**Figure S3**). On the other hand, FB_1_ had little impact on CEW larval growth at all concentrations tested (**Figure S3** and **Figure 4A**). In fact, at lower concentrations (below 30 ppm; **Figure S3**) the toxin seems to marginally enhance the growth of the larvae (the effect was consistent although there was variability among the bioassays). These results support our proposal that the enhanced infestation of *A. flavus* resistant maize lines by *H. zea* may be due to very low levels of AF that are not inhibitory to larval growth.

### Delayed Flowering in *A. flavus* Resistant Maize Lines

Tassel and ear development was delayed in the resistant inbred CML322 by 3 weeks relative to B73 and by 4–5 weeks in the resistant hybrid, Mp313E × Mp717 compared to GA209 × T173, although all four lines were planted together. CML322 is a tropical inbred and manifests delayed flowering under long days, i.e., ≥13 h photoperiod (Hung et al., 2012). The parents of the resistant hybrid (Mp313E × Mp717), derived from the tropical maize race Tuxpeño (Scott and Zummo, 1990; Williams and Windham, 2006), are also known to flower late. This is true for most maize lines that are resistant to *A. flavus*. Attempts to segregate the two traits, so far, have met with limited success (Henry, 2013). The availability of green silks may be an important factor for an increased *H. zea* infestation of often observed in the late flowering genotypes. However, in an adjacent plot where B73 was planted two weeks later (unrelated to the current study), silk emergence coincided with that of CML322 plants used in the present study. Nonetheless, B73 ears showed low levels of CEW infestation correlating with highly elevated levels of seed AF (400 ppb in controls and 800 ppb in inoculated plants) in this plot as well. These observations suggest that high seed AF levels suppress CEW infestation due to its toxicity, even if provision of green silks can promote CEW egg-laying.

## Discussion

The precise environmental factor that favored a CEW outbreak in 2018 is not clear. Unseasonal dry and warm weather is considered to support CEW population buildup in soybean but negatively affect infestation of drought-stressed maize (Herbert et al., 2003). For example, CEW damage was more severe in 2006 than in 2007 in the same maize field (Ni et al., 2011), although 2007 set the record as the then driest year in Georgia’s history (www.drought.gov/drought/states/georgia). Seasonal outbreaks of CEW population have been reported in the past but detailed correlative analysis between seasonal weather and CEW outbreaks is lacking. In a period of 25 years, severe crop damage by CEW was recorded during 9 of them in the state of Arkansas and no comparison to prevailing weather factors was made (reviewed in Dicke, 1939). There has also been no study where CEW infestation patterns have been compared in maize genotypes with varying resistance to *A. flavus* or AF accumulation, although toxicity of AF to CEW has been known for more than a decade (Zeng et al., 2006). Ni et al. (2011) compared spatial patterns of natural infestation of four ear-feeding insects including CEW with AF contamination by native *A. flavus* strains in a commercial maize hybrid. In the first year of the study, CEW infestation was extensive (95% of sampled ears) and in the second year, it was 41%. Although the low AF content observed in both years (>80% of ears had ≤30 ppb and only ≤4% ears had ≤100 ppb) makes it difficult to quantify the relationship between AF contamination and insect infestation, it indicated a negative association between CEW damage and seed AF content. The maize genotypes in our study have proven resistance or susceptibility to *A. flavus*. Further, high AF contamination (100 ppb) even in uninoculated susceptible lines has allowed to make robust comparisons.

The premise for this study is a novel and robust observation in that two unrelated maize lines (Tuxpeño germplasm versus CML) with proven resistance to *A. flavus* were heavily infested by CEW. Conversely, the two *A. flavus* susceptible lines (stiff-stalk inbred B73 and non-stiff stalk hybrid GA209 × T173) were spared by the pest. Although late flowering maize is known to be susceptible to CEW infestation by providing green silks, availability of silks alone could not fully explain our observations. Late flowering is more often a problem in the northeastern US where it coincides with CEW migration from southern states. Furthermore, late planted B73 in an adjacent plot had delayed silk emergence but showed no greater CEW infestation than the early planted set. The other and more likely explanation is that the susceptible lines had very high levels of AF that were toxic to CEW. Even mock-inoculated controls had 100 ng of AF per gram of seed meal prepared from entire ears that included both moldy and non-moldy seeds. This inference is supported by our feeding experiments (**Figure S3** and **Figure 4B**) as well as previous work (Zeng et al., 2006). Zeng et al. (2006) showed that AF at 200 ppb strongly inhibited the growth and development of first instar larvae, leading to >50% larval death after 9 d and 100% death after 15 d of feeding. Even lower concentrations (1-20 ppb; FDA-regulated levels) affected larval development, delayed pupation rate and led to >40% mortality when the exposure was longer than 7 d (Zeng et al., 2006). Although concentrations below 20 ppb were not tested in our study, we observed a steady decline in larval mass as AF concentration increased resulting in ≥30% mortality at or above 250 ppb during 10–15 d exposure (**Figure S3** and **Figure 4B**). We did not continue our observations beyond the larval stage to assess long-term developmental effects (e.g., pupation or emergence of adults). An apparent exception to the correlation between low AF and high CEW infestation was a significant decrease in CEW infestation observed in TOX4-inoculated ears compared to uninoculated ears in the *A. flavus* resistant inbred CML322, although average AF levels did not exceed 30 ppb. Given the highly variable distribution of AF in individual kernels of a maize ear (e.g., Lee et al., 1980), it is possible that the AF content particularly in damaged kernels at the ear tip was much greater than the average for the entire ear and high enough to be toxic to CEW. Furthermore, CEW may be sensitive also to other anti-insectan compounds that can be made by *A. flavus* (Cary et al., 2018) and act additively or synergistically with AF (e.g., Kojic acid; Dowd, 1988). Future experiments would involve late-maturing lines with *A. flavus* susceptibility and early maturing lines with *A. flavus* resistance to clarify and quantify the effects of flowering time and AF content on CEW infestation.

It is not surprising that AF is toxic to insects, not merely to mammals. *A. flavus* is predominantly a soil-living saprophyte, feeding on decaying organic matter, including dead insects. It is also an opportunistic pathogen and can colonize a wide variety of insects, e.g., moths, silkworms, bees, grasshoppers, houseflies, fruit flies and mealy bugs among others (St. Leger et al., 2000; Gupta and Gopal, 2002) and AF production may be an adaptive mechanism against fungivory (Drott et al., 2017). *A. flavus* is known to survive ingestion by mycophagous insects. Among three *Aspergillus* species tested, *A. flavus* conidia phagocytized by insect hemocytes were still able to germinate (St. Leger et al., 2000). *A. flavus* may also proliferate in the hindgut of CEW (Abel et al., 2002). Based on feeding studies in *Drosophila*, AF production is proposed to confer a fitness advantage to *A. flavus* when interacting with insects (Drott et al., 2017). In spite of being a polyphagous pest with a remarkable capacity to metabolize a wide array of plant compounds, CEW has limited tolerance to AF and poor ability to metabolize this mycotoxin (Dowd, 1988; Zeng et al., 2006). Beside AF, the fungus is known to make several anti-insectan compounds (TePaske et al., 1992; Cary et al., 2018). Other insect pests that are more tolerant may vector *A. flavus* (Zeng et al., 2006; Opoku et al., 2019; Reviewed in Munkvold et al., 2019). Based on spatial correlation analysis, Ni et al. (2011) reported that AF content was correlated to the frequency of weevils and stink bug-affected kernels, but not with CEW damage.

Our work also showed that FUM is not toxic to *H. zea* (**Figure S3** and **Figure 4A**). This may have allowed CEW to vector *F. verticillioides* and other FUM-contaminating fungi, as indicated by an increased seed FUM content in infested ears (**Figure 3**). CEW damage is also frequently associated with the colonization by another mycotoxigenic fungus, *Stenocarpella maydis*, which causes diplodia ear rot (Munkvold and White, 2016). In animal model systems, FB_1_ at 25-50 µM (i.e., 18-36 ppm) has been shown to inhibit ceramide synthases and lead to the accumulation of toxigenic/carcinogenic sphinganine and related compounds (Riley et al., 2001; Riley and Merrill, 2019). Conversely, FB_1_ was not found to be toxic even at 450 ppm to yellow mealworm larvae when included in the diet or injected into larva (Abado-Becognee et al., 1998). Recently, the brown marmorated stink bug (*Halyomorpha halys*) was shown to enhance *F. verticillioides* infection and FUM contamination in field corn (Opoku et al., 2019). Among other secondary metabolites produced by *F. verticillioides*, fusaric acid is only a weak antisectan compound (Dowd, 1988). The lack of secondary metabolites with potent insecticidal properties in the biosynthetic repertoire of *F. verticillioides* could be one of the reasons for its frequently observed transmission *via* insect infestation (e.g., Smeltzer, 1959; Dowd, 2000; Alma et al., 2005; Mesterházy et al., 2012; Madege et al., 2018) and a critical link between insect damage and Fusarium ear rot (Munkvold et al., 2019). Successful mitigation of mycotoxins requires control of multiple pests, including CEW (Abbas et al., 2013; Bowers et al., 2014; Porter and Bynum, 2018). Bt-maize has been highly successful in crop protection from important pests, including CEW. However, global warming has been shown to enhance the risks of extensive Bt-adaptation as well as overwintering of CEW in the northern US (Venugopal and Dively, 2017) and could exacerbate the mycotoxin problem.

Although this study was pursued to explain a serendipitous observation, it has important implications in mycotoxin control. AF and FUM are ubiquitous and unpredictable contaminants of commodities, particularly maize. Our study clarifies a component of this unpredictability. The late flowering trait of *A. flavus* resistant lines (owing to their tropical origin) is known to delay harvest, potentially leading to frost damage and/or high grain moisture. Our current work shows that delayed flowering when coupled with unseasonal weather and low AF accumulation can exacerbate CEW infestation, which in turn can lead to contamination by other mycotoxins, such as fumonisins (Munkvold and White, 2016).

In contrast to a mutual antagonism reported previously between *A. flavus* and *F. verticillioides* (Zummo and Scott, 1992; also see **Figure S4**), we observed high levels of AF and FUM co-contaminating our samples. B73, in particular with its high susceptibility to both mycotoxigenic fungi, had very high levels of both AF and FUM in many of its seed samples. Although CEW damage was very low in this inbred (**Figures 1B** and **2**), FUM levels were exacerbated in infested ears (**Figure 3B**). There is some evidence for an additive or even synergistic effect on carcinogenicity from co-exposure to AF and FUM (World Health Organization, 2018). Based on biomarker studies and food analyses, the co-occurrence of these two mycotoxins has been widely documented in developing countries (Shirima et al., 2013; Biomin, 2019). It is important to examine the underlying factors as well as effects of mycotoxin co-contamination both by researchers and regulatory agencies to mitigate its impact on food safety (Lopez-garcia, 1998). As demonstrated by our study, a host genotype even with demonstrable resistance can become vulnerable due to seasonal variation in flowering time or an outbreak of chewing insects. Further, incorporation of resistance to a single mycotoxin accumulation and not pairing it with insect resistance may not adequately ensure food safety.

## Data Availability Statement

The raw data supporting the conclusions of this article will be made available by the authors, without undue reservation.

## Author Contributions

SC planned the study. SC, MB, and FH carried out the experiments. WW, JH contributed resources. SC assembled and analyzed the data, and wrote the manuscript. All authors contributed to the article and approved the submitted version.

## Funding

SC thanks the National Corn Growers Association for the funding support through their AMCOE program.

## Conflict of Interest

The authors declare that the research was conducted in the absence of any commercial or financial relationships that could be construed as a potential conflict of interest.
