# Supplementary material for: Low Aflatoxin Levels in Aspergillus flavus-Resistant Maize Are Correlated With Increased Corn Earworm Damage and Enhanced Seed Fumonisin
Source: Front Plant Sci. 2020 Sep 23;11:565323. doi: 10.3389/fpls.2020.565323 (PMC7546873; doi:10.3389/fpls.2020.565323)
Supplement: Supplementary file 1 [file DataSheet_1.pdf]

frontiers

frontiers

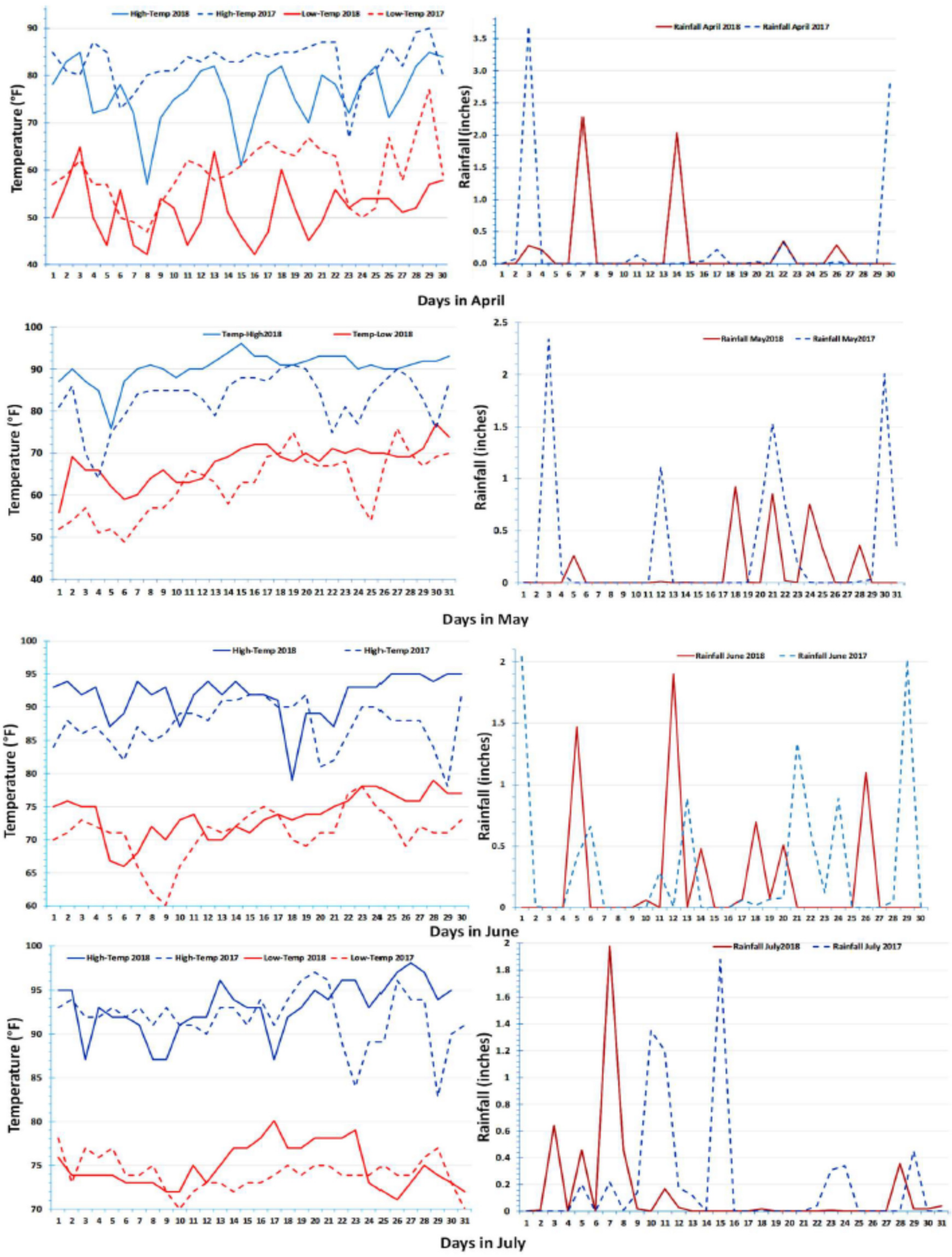

Figure S1. Weather data for the months of April-July in 2018 (solid lines) and 2017 (dashed lines). Daily high (blue lines) and low (red lines) temperatures are shown in the left panel. Rain fall is shown in the right panel.

SUPPLEMENTARY FIGURE 1. Weather data for the months of April-July in 2018 (solid lines) and 2017 (dashed lines). Daily high and low temperatures (red and blue lines respectively) are shown in the left panel. Rain fall is shown in the right panel.

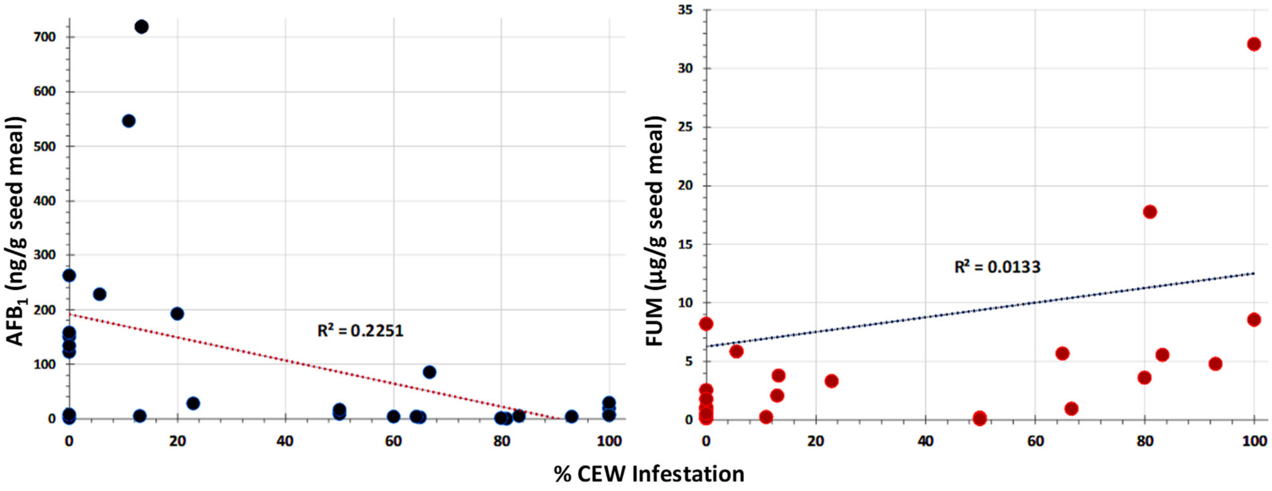

SUPPLEMENTARY FIGURE 2. Correlation between CEW infestation of ears and seed AF or FUM levels in maize. Combined data from inbred and hybrid maize lines is plotted. CEW showed a negative relationship with AF and a positive trend with FUM. The greater correlation observed with AF (Pearson correlation coefficient, R = -0.47) was likely because of manual inoculation with specific strains of *A. flavus* (dominant to native strains), whereas more random infestation by native *Fusarium* strains may have led to poor correlation (R = 0.115).

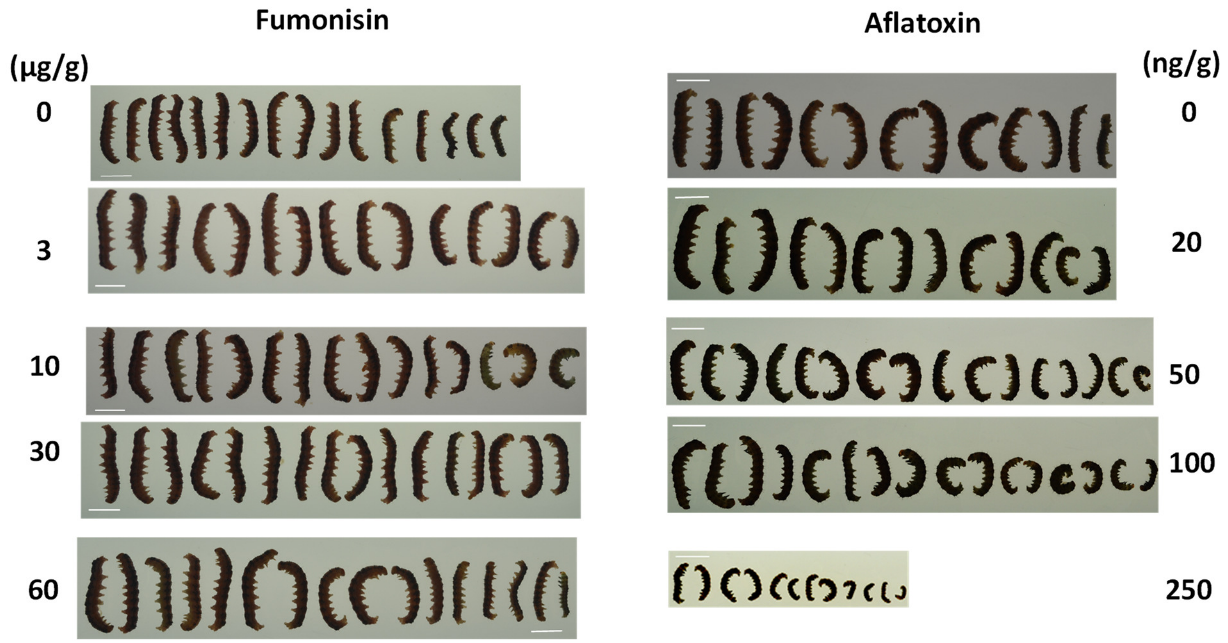

SUPPLEMENTARY FIGURE 3. **Effects of aflatoxin B_1 and fumonisin B_1 on the growth, mortality and body mass of *H. zea* larvae.** Graded doses of FB_1 (Left panel) and AF (Right panel) were tested on CEW growth and mortality by incorporating them into an artificial insect diet. Larvae were grown in a 128 well bioassay plate for 10 d. Each well had 1 g of feed and a single neonate at the start of the assay. A representative assay from 4 replicated experiments is shown. In an additional assay, 100 ppm of FB_1 and 300 ppb of AF were tested. Results were not different, except for a greater larval mortality at 300 ppb of AF (data not shown). Scale Bar = 1 cm.

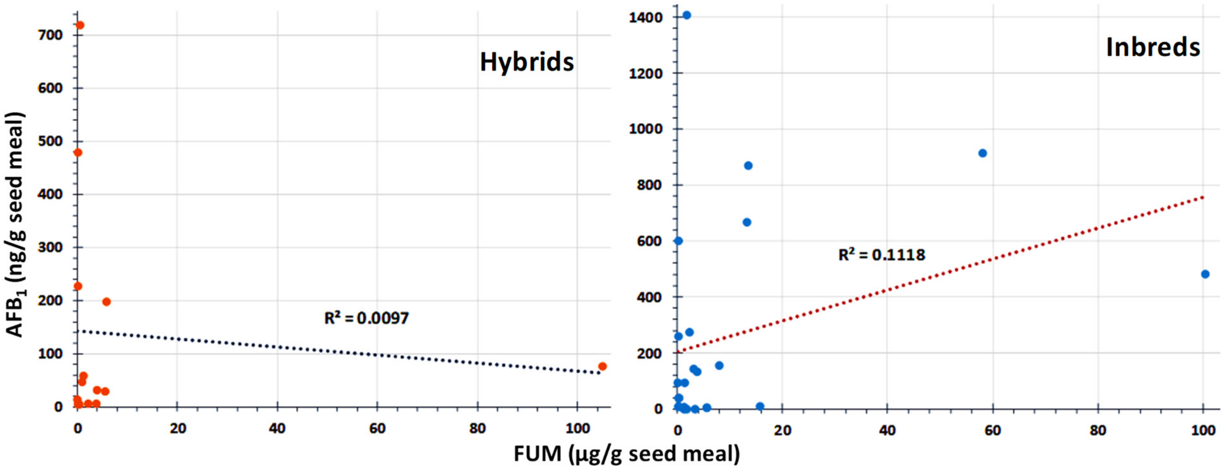

SUPPLEMENTARY FIGURE 4. Correlation of Seed FUM and AF contents in hybrids and inbreds. Contents of the two mycotoxins from the same seed sample are poorly correlated in both sets as indicated by Pearson correlation coefficient values (r = -0.0983 for hybrids and 0.3344 for inbreds). This lack of correlation indicated that there was no mutual effect in the production of the two mycotoxins by the fungi infecting seeds from same ears.

TABLE S1. Analysis of variance (ANOVA) and post hoc analysis with Tukey's Test for CEW infestation in maize inbreds and hybrids with contrasting resistance to aspergillus ear rot. Tukey's test results are shown only for groups that are significantly different in ANOVA.

	Df	Sum Sq	Mean Sq	F value	Pr(>F)	
Infection	1	693	693	1.595	0.225	
Genotype	3	20828	6943	15.970	4.54e-05	***
Infection:Genotype	3	321	107	0.246	0.863	
Residuals	16	6956	435			
Residuals	16	2250	141			

Signif. code: 0 '***'

Tukey's HSD

$Genotype	diff	lwr	upr	p adj
SusHybrid-ResHybrid*	-68.27	-102.71	-33.83	0.000
SusInbred-ResInbred*	-46.50	-80.94	-12.06	0.007

*ResHybrid = Resistant hybrid, Mp313E×Mp717; SusHybrid = Susceptible hybrid, GA209×T173; ResInbred = Resistant inbred, CML322; SusInbred = Susceptible inbred, B73

TABLE S2. ANOVA and Tukey's post hoc test for seed AF content in maize lines with contrasting resistance to aspergillus ear rot and CEW infestation rates. Tukey's test results are shown only for groups that are significantly different in ANOVA.

	Df	Sum Sq	Mean Sq	F value	Pr(>F)	
Genotype	3	3623512	1207837	34.373	3.99e-10	***
Infection	1	726043	726043	20.662	7.41e-05	***
Infestation	1	308549	308549	8.781	0.005705	**
Genotype:Infection	3	765751	255250	7.264	0.000753	***
Genotype:Infestation	3	423363	141121	4.016	0.015605	*
Infection:Infestation	1	1979	1979	0.056	0.813916	
Genotype:Infection :Infestation	3	125570	41857	1.191	0.328698	
Residuals	32	1124456	35139			

Signif. codes: 0 '***' 0.001 '**' 0.01 '*' 0.05

Tukey's HSD

$Genotype	diff	lwr	upr	p adj
SusHybrid-ResHybrid	313.16	105.81	520.50	0.001
SusInbred-ResInbred	667.28	459.94	874.62	0.000

$Infection				
Infected-Control	245.98	135.75	356.20	0.000

$Infestation				
Uninfested-Infested	160.35	50.13	270.58	0.006

$Genotype:Infection	diff	lwr	upr	p adj
ResHybridInfected-ResHybridControlF*	-13.38	-363.96	337.20	1.000
SusHybridInfected-ResHybridControlF	503.96	153.38	854.54	0.001
SusInbredControlF-ResInbredControlF	384.71	34.13	735.29	0.023
ResInbredInfected-ResInbredControlF	18.58	-332.00	369.16	1.000
SusInbredInfected-ResInbredControlF	968.43	617.85	1319.01	0.000
ResHybridInfected-SusHybridControlF-	122.35	-472.93	228.23	0.945
SusHybridInfected-SusHybridControlF	394.99	44.41	745.57	0.018
ResInbredInfected-SusInbredControlF-	366.14	-716.72	-15.56	0.036
SusInbredInfected-SusInbredControlF	583.71	233.13	934.29	0.000
SusHybridInfected-ResHybridInfected	517.34	166.76	867.92	0.001
SusInbredInfected-ResInbredInfected	949.85	599.27	1300.43	0.000

$Genotype:Infestaton`				
SusHybridInfested-ResHybridInfested	211.67	-138.91	562.25	0.525
ResHybridControlI*-ResHybridInfested	-11.10	-361.68	339.47	1.000
SusHybridControlI-ResHybridInfested	403.53	52.95	754.11	0.015
SusInbredInfested-ResInbredInfested	442.63	92.05	793.21	0.006
ResInbredControlI-ResInbredInfested	5.67	-344.91	356.25	1.000
SusInbredControlI-ResInbredInfested	897.61	547.03	1248.19	0.000
ResHybridControlI-SusHybridInfested	-222.78	-573.36	127.81	0.461
SusHybridControlI-SusHybridInfested	191.86	-158.72	542.44	0.642

Table S2, continued..,

$Infection:Infestation

	diff	lwr	upr	p adj
ControlF&ControlI-ControlF&Infested	173.19	-34.15	380.54	0.128
Infected&ControlI-ControlF&Infested	406.33	198.98	613.67	0.000
ControlF&ControlI-Infected&Infested	-85.62	-292.97	121.72	0.681
Infected&ControlI-Infected&Infested	147.51	-59.83	354.85	0.237
Infected&ControlI-ControlF&ControlI	233.13	25.79	440.47	0.023

*ControlF = Ears that were not inoculated by the fungus, *A. flavus*; ControlI = Ears that were not infested by the insect, *H. zea*
